# Different effectiveness of closed embryo culture system with time-lapse imaging (EmbryoScope^TM^) in comparison to standard manual embryology in good and poor prognosis patients: a prospectively randomized pilot study

**DOI:** 10.1186/s12958-016-0181-x

**Published:** 2016-08-24

**Authors:** Yan-Guang Wu, Emanuela Lazzaroni-Tealdi, Qi Wang, Lin Zhang, David H. Barad, Vitaly A. Kushnir, Sarah K. Darmon, David F. Albertini, Norbert Gleicher

**Affiliations:** 1 The Center for Human Reproduction, 21 East 69th Street, New York, NY 10021 USA; 2 The Foundation for Reproductive Medicine, New York, NY 10021 USA; 3 Department of Obstetrics and Gynecology, Albert Einstein College of Medicine, Bronx, NY 10461 USA; 4 Department of Obstetrics and Gynecology, Wake Forest University, Winston Salem, NC 27106 USA; 5 Department of Molecular and Integrative Physiology, The University of Kansas School of Medicine, Wichita, KS 64109 USA; 6 Stem Cell Biology and Molecular Embryology Laboratory, The Rockefeller University, New York, NY 10065 USA

**Keywords:** In vitro fertilization (IVF), Time laps photography, Closed incubation systems, Embryology, Embryo quality, Cost-effectiveness

## Abstract

**Background:**

Previously manual human embryology in many in vitro fertilization (IVF) centers is rapidly being replaced by closed embryo incubation systems with time-lapse imaging. Whether such systems perform comparably to manual embryology in different IVF patient populations has, however, never before been investigated.

We, therefore, prospectively compared embryo quality following closed system culture with time-lapse photography (EmbryoScope™) and standard embryology.

We performed a two-part prospectively randomized study in IVF (clinical trial # NCT92256309). Part A involved 31 infertile poor prognosis patients prospectively randomized to EmbryoScope™ and standard embryology. Part B involved embryos from 17 egg donor-recipient cycles resulting in large egg/embryo numbers, thus permitting prospectively alternative embryo assignments to EmbryoScope™ and standard embryology.

We then compared pregnancy rates and embryo quality on day-3 after fertilization and embryologist time utilized per processed embryo.

**Results:**

Part A revealed in poor prognosis patients no differences in day-3 embryo scores, implantation and clinical pregnancy rates between EmbryoScope™ and standard embryology. The EmbryoScope™, however, more than doubled embryology staff time (*P* < 0.0001). In Part B, embryos grown in the EmbyoScope™ demonstrated significantly poorer day-3 quality (depending on embryo parameter between *P* = 0.005 and *P* = 0.01). Suspicion that conical culture dishes of the EmbryoScope™ (EmbryoSlide™) may be the cause was disproven when standard culture dishes demonstrated no outcome difference in standard incubation.

**Conclusions:**

Though due to small patient numbers preliminary, this study raises concerns about the mostly uncontrolled introduction of closed incubation systems with time lapse imaging into routine clinical embryology. Appropriately designed and powered prospectively randomized studies appear urgently needed in well-defined patient populations before the uncontrolled utilization of these instruments further expands.

**Trial registration:**

NCT02246309 Registered September 18, 2014.

## Background

Since its inception, improvements in the embryology laboratory have been a consisten goal of in vitro fertilization (IVF). Over the years this has led to significant changes in how human embryos are processed: New culture media have been introduced [1, 2]; length of embryo culture has in many IVF centers changed from 2–3 days to 5–6 days) [3, 4]; and culture at reduced oxygen tension has been reported to improve embryo development and clinical pregnancy rates [5, 6]. Increasingly, there has also been talk in recent years about automating embryology, whether to improve quality or cost effectiveness [7–9].

It, therefore, was no surprise when in recent years a series of automated closed incubation systems became commercially available. They offered the additional benefit of allowing consistent observation of embryo development via time-lapse imaging (TLI), without need of opening incubator doors, reported to be detrimental to embryos [10, 11].

By minimizing environmental fluctuations in temperature, pH and humidity, supporters of such closed incubation systems have argued that embryo quality would be improved and, therefore, clinical outcomes. They also suggested that continuous time-lapse documentation of embryo development would improve embryo selection and, thereby, further improve pregnancy chances [12–16]. These instrumentations, however, entered the market place without clinical validation for either claim. Till today it, therefore, is unknown whether embryos from different patient populations (ie, good vs. poor prognosis or younger vs, older patients) are affected differently by such culture systems.

In traditional embryology, embryo selection is performed manually and individually, trying to remove embryos from controlled incubation only as rarely as possible. Embryo assessment is, therefore, dependent on experience and training of embryologists, and will vary [8].

Time-lapse imaging (TLI) systems, in contrast, offer computer assisted, objective and non-invasive embryo assessments. Continuous recording of all key developmental events, at least hypothetically, allows for improved embryo selection based on observations, which would be missed by manual embryology. They, therefore, have been alleged to improve embryo selection and clinical pregnancy chances [17]. Several studies have claimed early timing events to be predictive of blastocyst formation [12, 18, 19], implantation [20, 21] and pregnancy [20, 22]. Currently available data appear, however, insufficient to support the conclusion that TLI systems, indeed, are helpful enough in embryo selection to improve IVF cycle outcomes [11].

Several recent studies of TLI systems failed to achieve improvements in embryo quality and other clinical outcomes [23–26]. Park et al., indeed, reported a significant increase in miscarriages after TLI [26].

Most so far reported studies of TLI have been anecdotal and uncontrolled. To the best of our knowledge, only two prospectively randomized studies have so far been reported [27, 28], one in very good prognosis patients reporting a marginal benefit for TLI [27] and the other [28] reporting no difference to standard embryology. Three systematic reviews also concluded that there was no outcome benefit from TLI [11, 29, 30]. Moreover, IVF interventions that may be successful in good prognosis patients, may lack benefits in other patient populations, like average-prognosis patients, and have been demonstrated to be actually detrimental to outcomes in poor-prognosis patients [31].

We, therefore, in this study attempted to asses the clinical value of a TLI system in two distinct patient populations: We, first, prospectively investigates a group of poor-prognosis patients by randomizing them to TLI and standard embryology. Secondly, we, however, investigated a group of egg donors as best-prognosis patients.

As this study will demonstrate, TLI systems in human embryology laboratories requires careful evaluation before further integration into routine human IVF practice since their effectiveness appears to be patient-dependent.

## Methods

### Institutional review board (IRB)

Since the EmbryoScope™ was approved by The Food and Drug Administration (FDA) for use in human embryology laboratories, and has been integrated into routine embryology practice in many IVF centers, here reported clinical trial (clinical trial registration # NCT02256309, available at http://www.ClinicalTrials.gov) represented a prospective clinical comparison of two standard embryology practices in IVF, and was, therefore, approved by our center’s IRB by expedited review. Patients signed an appropriate informed consent.

### Primary and secondary outcome measures

Primary outcome was clinical pregnancy rate per IVF cycle start, with a clinical pregnancy being defined as a pregnancy visualized by ultrasound and demonstrating a normal fetal heart rate. Secondary outcomes were embryologist time spent per embryo observation and embryo quality.

### Patient selection and randomization

Here reported study was restricted to 3.5 months beween December 2014 and March 2015, the time period our center (Center for Human Reproduction in New York City) was offered free use of an EmbroScope™ by the manufacturere. A follow-up study, which attempted to determine potential causes for observed findings in Part B of the study, was carried out between April and June, 2015.

Part A of the study was an open-label prospectively randomized clinical trial, offered during the study period to consecutive patients undergoing autologous IVF (+ICSI) cycles. The primary study outcome was clinical pregnany. Anticipation was to recruit approximately 30 patients per month, resulting in a study population for randomization of ca. 100 patients. We initiated the study as a pilot study since power analysis suggested that the expected patient number to detect at least 20 % difference in clinical pregnancy rates would, likely, be insufficient. Our hope was that the manufacturer might extend our access to the equipment beyond 3 months to reach required numbers for statistical power. Power analyses of secondary outcomes, including embryo parameters, and time analysis of embryologists, suggested need for fewer patients/ cycles to reach statistically valuable information. We, therefore, assumed that without extended availability of the instrument, we still should be able to assess potentially important secondary outcomes with adequate statistical power.

**Fig. 1:**
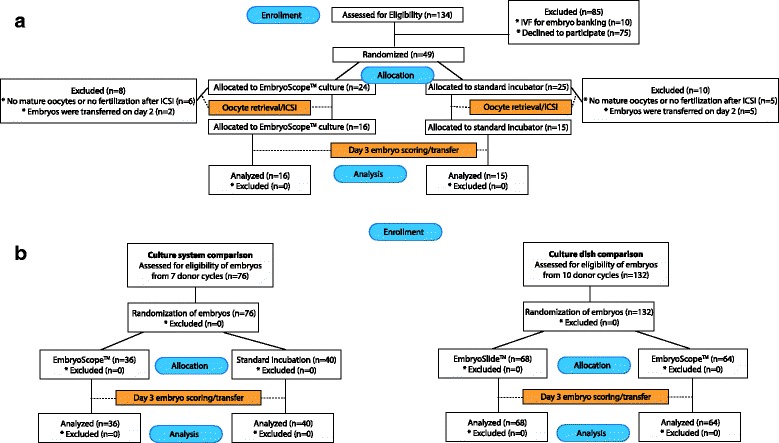
Consort flow diagram for the TLI culture randomized control trial (ClinicalTrials.gov NCT02256309)

Figure 1 summarized the CONSORT flowchart for this trial: In part A of the trial 134 patients were offered participatation during the study period. To our surprise only 49 qualified and/or consented to participate by informed consent. Computer randomization to either TLI or standard embryology was the responsibility of a member of the center’s Statistics Section (SKD) who was completely disassociated from the patient’s IVF cycle. The designation was then reported to the embryology staff, which processed the patient’s oocytes/embryos accordingly.

Related to our center’s adverse patient selection, the final number of women undergoing randomization for part A was only 31 (Panel a) after exclusion of other patients. Table 1 demonstrates that, based on age and/or functional ovarian reserve parameters (FSH and AMH), they, indeed, were poor-prognosis patients. As such, they also, as expected, produced relative small oocyte and embryo numbers, prohibiting inter-embryo randomization, and mandating transfer of all transferrable embryos. Panel b describes part B of the study, where embryos in donor recipient cycles were randomized prospectively rather than patients.

**Table 1:** Patient and cycle characteristics of study Part A

IVF information and characteristics	EmbryoScope^TM^ (*N* = 16)	Standard (*N* = 15)	*P* value
Average age (years)	38.8 ± 1.0	40.4 ± 1.8	0.65
Average serum FSH (mIU/ml)	9.2 ± 1.2	10.8 ± 1.6	0.46
Average serum AMH (ng/ml)	1.1 ± 0.5	0.9 ± 0.3	0.74
Number of oocytes/patient (n)	5.3 ± 0.9	4.4 ± 0.7	0.52

*N* number of patients

Such inter-embryo randomization among individual patients is, however, possible in young egg donors, who routinely produce large oocyte and embryo numbers. In Part B of this study, we, therefore, separated during the study period egg donor cycles from autologous IVF cycle. Moreover, egg donors were not randomized but, within each donor egg cycle, alternating embryos were either assigned to TLI or standard embryology. This part of the study, thus, prospectively assigned 76 embryos from 7 oocyte donor/recipient cycles (CONSORT flow chart in Fig. 1b), and the assignment was done in non-blinded fashion by a senior embryologist. Table 2 summarizes patient characteristics of Part 2 patients (oocyte donors).

**Table 2:** Patient (oocyte donors) and cycle characteristics of study Part B

IVF information and characteristics	EmbryoScope^TM^ vs. Standard (*N* = 7)	EmbryoSlideTM vs. Standard dish (*N* = 10)
Average age (years)	27.8 ± 1.4	26 ± 0.9
Average serum AMH (ng/ml)	4.3 ± 1.1	6.1 ± 0.9
Number of oocytes/donor (n)	17.3 ± 2.8	19.3 ± 1.8

*N* number of patients

#### Assessment of the EmbryoSlide™

Since results of Part B of the study suggested significant outcome differences between TLI and standard embryology, we wanted to determine whether the conical culture dish in the EmbryoScope™ (EmbryoSlide™) might be the culprit. The hypothesis was that embryos may be incubating at the bottom of the conical well, surrounded by excretion products, which might adversely affect embryo quality.

The assessment of this hypothesis was no longer dependent on availability of the EmbryoScope™. This study was, therefore, extended by approximately 3 months to determine whether the conical form of the EmbryoSlide™ was, indeed, the culprit. We had purchased excessive numbers of EmbryoSlides™ for the clinical trial of the EmbryoScope™, which now were used prospectively. Ten additional donor-recipient cycles (132 embryos) were utilized to culture embryos in standard incubators, alternating in EmbryoSlides™ and flat standard culture dishes, our center routinely uses.

Our center implements changes in the embryology laboratory’s routine only very cautiously. Before implementing a change, the laboratory’s routine is compared prospectively to the potentially new product/procedure. The center’s Institutional Review Board (IRB) does not require prior review of such comparisons if alternative products/procedures reflect standard of care and/or have received approval from the Food and Drug Administration since such investigations are considered part of the laboratory’s continous quality improvement program.

This part of the study, therefore, did not undergo IRB review.

### Patient stimulation and IVF procedure

All subjects underwent controlled ovarian hyperstimulation and oocyte maturation by human chorionic gonadotropin (hCG), followed by transvaginal ultrasound-guided oocyte retrieval. hCG was administered when leading follicles reached 19–21 mm. Oocyte donors were stimulated in a long gonadotropin releasing hormone agonist cycle (GnRHa, Lupron, leuprolide acetate, Takeda Pharmaceutical U.S.A Inc) with daily dosages of 150–300 I.U. of human menopausal gonadotropin (hMG) from various manufacturers. In contrast, infertility patients were stimulated in microdose agonist cycles (Lupron) with daily dosages of 450–600 I.U. of gonadotropins, typically in a majority (300–450 I.U.) administered as follicle stimulating hormone (FSH), and in a minority (150I.U.) as hMG.

All media and reagents for IVF were purchased from LifeGlobal (Guilford, CT, USA) unless indicated otherwise below. For blastocyst culture their Global®Total® media was used. Oocyte-cumulus complexes (COCs) were collected using transvaginal ultrasound guided follicle punctures in mHTF, containing 6 % human serum albumin (HSA). Before ICSI, COCs were cultured in HTF, containing 10 % HSA in an organ dish (Falcon, VWR, NJ, USA). After removal of cumulus by 30 s of hyaluronidase treatment, oocytes were assessed according to morphology. Oocytes with obvious first polar body (1st Pb) were identified as mature (MII), and used for ICSI. ICSI was performed under an inverted microscope (Olympus, Japan) within six hours of oocyte retrieval in mHTF, containing 6 % HAS.

### Embryo culture

#### Standard embryology

For the standard embryo culture group, oocytes after ICSI were washed in blastocyst medium containing 10 % HSA, and then transferred to pre-equilibrated culture dish (Thermo, Oskilde, Denmark), with 20 μl droplets of blastocyst medium containing 10 % HSA under light oil. The embryos were cultured until transfer in a standard incubator (Panasonic, Japan) at 37 °C, 5 % CO_2_ and 90 % N_2_ for 3 days. The embryos were taken out of the incubator at 16–18 h post injection for fertilization check, at 40–42 h post ICSI for early cleavage evaluation, and at 64–66 h post ICSI for quality assessment of transfer and cryopreservation. The time used for each check and assessment was recorded.

#### TLI embryology

For TLI embryo culture group (EmbryoScope^TM^), oocytes were washed in the same way as described above for the standard embryology group. We utilized in this study the EmbryoScope^TM^ (Vitrolife, Göteborg, Sweden), a commercial, FDA-approved TLI system. It is made up of an incubator with a built-in microscope, which acquires images of cultured embryos continuously. In this study, we acquired images every 10 min at seven focal planes.

Pre-equilibrated embryo culture dishes (EmbryoSlide™, Vitrolife, Göteburg, Sweden) were used in conjuction with the EmbryoScope^TM^, which were prepared by following the manufacturer’s instruction. Briefly, each well contained 25 μl of Blastocyst medium containing 10 % HAS, and the whole dish was covered with 1.5 ml of light oil. After ICSI, individual oocytes were loaded to the center of the well and cultured in the EmbryoScope^TM^ at 37 °C, 5 % CO_2_, %5 O_2_ and 90 % N_2_ atmosphere for 3 days until transfer. The images taken by the TLI were reviewed at 16–18 h post injection for fertilization check, at 40–42 h post ICSI for early cleavage evaluation, and at 64–66 h post ICSI for embryo quality assessment for embryo transfer and cryopreservation. The embryology staff time used for each check and assessment was recorded.

### Assessment of embryology staff time

The time embryologists used on either embryology method was timed by a standard lab timer. For standard culture, timing was taken from when a culture dish was removed from the incubator until the dish was returned back into the incubator. For TLI culture, timing was started when the embryologist began to check a first embryo’s morphology and discontinued with completion of the last embryo’s check. To avoid the subjective difference of different observers, all embryo checking/selection and time recording were performed only by one embryologist.

### Comparison of standard embryo culture dish and EmbryoSlide™

Oocytes were loaded as described above. Comparing out center’s standard culture dish to EmbryoSlide™, both were prepared as described before, were and injected and were cultured in a standard incubator at 37 °C, 5 % CO_2_ and 90 % N_2_ atmosphere for 3 days until transfer. Embryos in both dishes were taken out of the incubator at 16–18 h post injection for fertilization check, at 40–42 h post ICSI for early cleavage evaluation, and at 64–66 h post ICSI for embryo quality assessment for embryo transfer and cryopreservation.

### Embryo assessment and selection on day 3

For all groups of embryo in this study, morphological assessments and selection for embryo transfer were performed at the same time point (64–66 h post ICSI) and using the same criteria. Additional information from TLI was not considered for embryo assessment and selection.

**Fig. 2:**
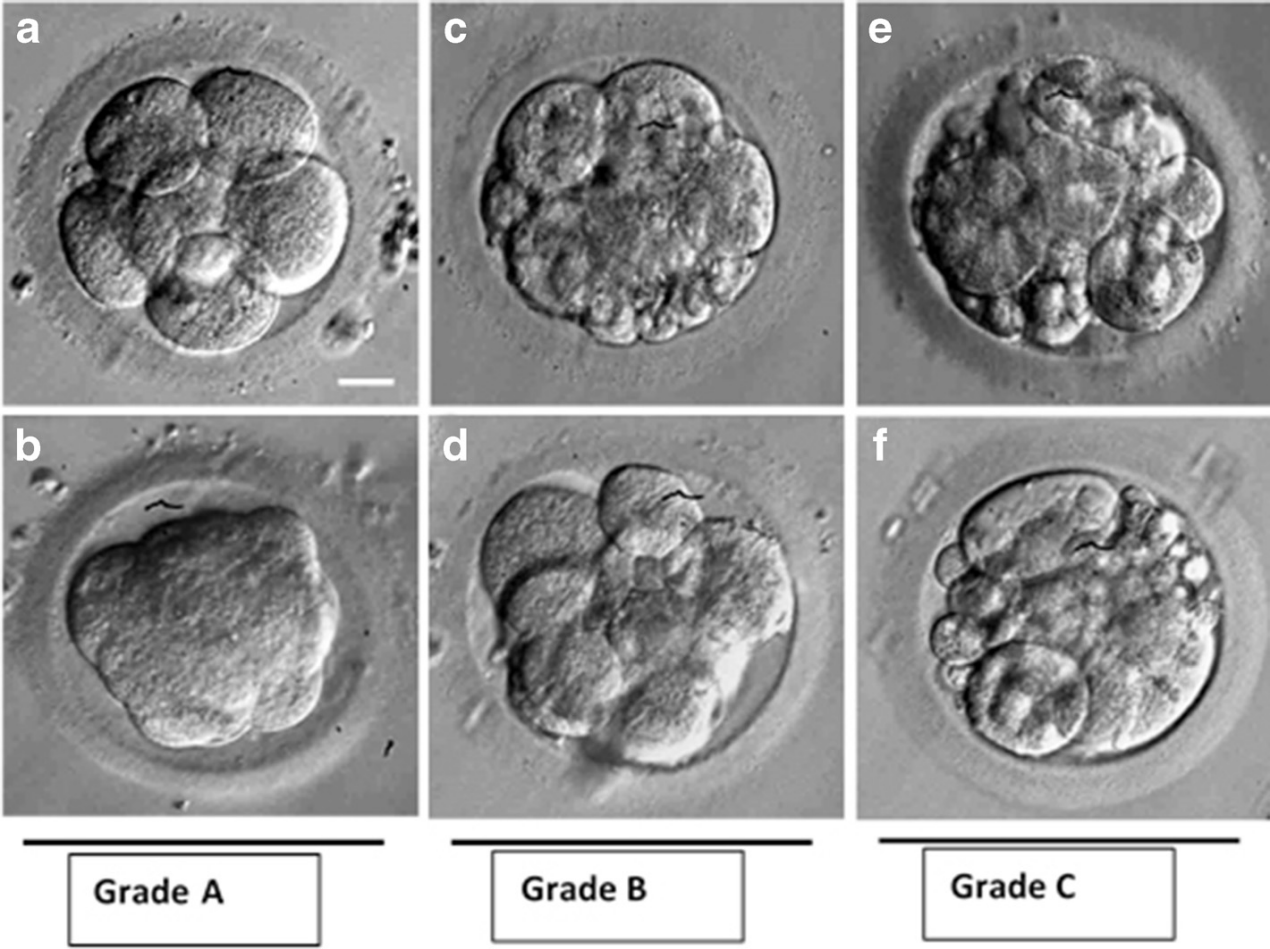
Grades for human embryo of day 3 cultured in vitro. Panels **a** and **b** (Grade A) demonstrate best quality embryos; Panels **c** and **d** depict intermediate grade embryos (Grade B); and Panels **e** and **f** show worst grade embryos (Grade C)

On day 3, at 64–66 h post ICSI, embryos were scored according to blastomere numbers, size and amount of fragmentation. Embryos of Grade A (high quality) had ≥ 8 blastomeres with equal size, <10 % fragmentation or slightly unequal size and no fragmentation; Embryos of Grade B (fair quality) had ≥ 6 blastomeres with equal size, <25 % fragmentation or slightly unequal size and <10 % fragmentation; Embryos of Grade C (poor quality) had > 25 % fragmentation or blastomeres with severely unequal size. Examples for embryo grading criteria are demonstrated in Fig. 2. Embryos of grade A and B were considered suitable for transfer or cryopreservation.

All embryo transfers were carried out on day-3, within 2 h from the embryo quality assessment. Panels a and b (Grade A) demonstrate best quality embryos; Panels c and d depict intermediate grade embryos (Grade B); and Panels e and f show worst grade embryos (Grade C).

### Statistical analysis

All statistical analyses were performed using Prism software (GraphPad Prism 6.0, GraphPad Software. Inc, CA, USA). The unpaired *t*-test with Welch’s correction was used for all statistical comparisons. Data in all tables are shown as value ± SEM. Values were considered statistically significant at *P* < 0.05.

## Results

### Part A: TLI system vs. standard embryo culture system system in infertile women

#### Patient comparisons

Randomization of 31 infertile patients resulted in 16 patients going through TLI and 15 through standard embryology (Fig. 1a). Patient and IVF cycle characteristics involved in this study are summarized in Tables 1 and 2: Mean age in the EmbryoScope^TM^ group (38.8 ± 1.0 years) was non-significantly lower than in the standard embryology group (40.4 ± 1.8 years). Similarly, FSH, AMH and number of retrieved oocytes (5.3 ± 0.9 vs. 4.4 ± 0.7) did not differ, suggesting a credible randomization process.

#### Pregnancy rates

A total of 44 fertilized oocytes from 16 patients were cultured in the EmbryoScope^TM^, and 42 fertilized oocytes from 15 patients were cultured in standard incubators. Embryo numbers per patient cultured in EmbryoScope^TM^ and standard incubators were similar (2.7 ± 0.4 vs. 2.8 ± 0.4). No differences were also noted in number of good quality embryos, defined as Grade A on day 3 (1.2 ± 0.3 vs. 1.2 ± 0.2), fair quality embryos, Grade B on day 3 (0.9 ± 0.2 vs. 0.9 ± 0.3) and poor quality embryos (Grade C on day 3; 0.4 ± 0.2 vs. 0.6 ± 0.2) (Table. 3). The clinical pregnancy rate per randomized patient was 18.8 % in the EmbryoScope^TM^ group and 20.0 % in the standard embryology group, and implantation rates were 9.7 and 11.5 %, respectively. Considering the adverse selection of patients in Part A, these IVF cycle outcomes have in both groups to be considered as respectable, though not remarkable.

**Table 3:** Outcome comparisons in study Part A

Embryo development and outcomes	EmbryoScope^TM^ (*N* = 16)	Standard (*N* = 15)	*P* value
Number of Total embryos per patient (n)	2.7 ± 0.4	2.8 ± 0.4	0.93
Number of Grade A embryos per patient (n)	1.2 ± 0.3	1.2 ± 0.2	>1.0
Number of Grade B embryos per patient (n)	0.9 ± 0.2	0.9 ± 0.3	>1.0
Number of Grade C embryos patient (n)	0.4 ± 0.2	0.6 ± 0.2	0.99
Pregnancy rate (%)	18.8	20.0	>1.0
Implantation rate (%)	9.7	11.5	>1.0

Since this study involved poor prognosis patients, all transferrable embryos obtained in a cycle were transferred

*N* number of patients

In a population of relative poor prognosis patients, embryos cultured in a TLI system and by standard embryology up to day-3, thus, demonstrated similar development and similar implantation as well as clinical pregnancy rates. The small number of investigated patients, however, does not preclude the possibility of a type 2 error. In other words, this study does not preclude the possibility that a larger patient population might demonstrate significant differences between TLI and standard embryology. It in this context is important to note that all non-significantly different outcomes did trend in favor of standard embryology.

#### Time study

Table 4 demonstrates that, in contrast to clinical outcomes, staff time both embryo culture systems required, significantly differed: The EmbryoScope^TM^ more than doubled the required transaction time (301.2 ± 80.8 s) per embryo compared to standard embryology (137.6 ± 2.7 s; *P* < 0.0001).

**Table 4:** Comparison of embryologists’ time usage in study Part A

Time usage for embryo check per embryo (seconds)	EmbryoScope^TM^ (*N* = 16; *n* = 44)	Standard (*N* = 15; *n* = 42)	*P* value
Day 1 (fertilization check)	99.0 ± 34.1	49.8 ± 17.0	*P* < 0.0001
Day 2 (Cleavage check)	98.6 ± 32.2	47.8 ± 14.9	*P* < 0.0001
Day 3 (Selection for transfer)	103.6 ± 23.9	54.1 ± 17.9	*P* < 0.0001
Total	301.2 ± 80.8	137.6 ± 52.7	*P* < 0.0001

*N* number of patients, *n* number of embryos

### Part B. TLI system vs. standard embryo culture system in young oocyte donors

Table 5 summarizes embryo grades of 7 oocyte donors whoes oocytes/embryos were randomly assigned to either the EmbryoScope™ or to the center’s standard embryology. Among 36 embryos cultured in the EmbryoScope^TM^, 55.8 ± 6.4 % were of grade A, a significantly lower percentage than achieved with standard culture (81.2 ± 4.1 % of 40; *P* = 0.005). Moreover, there were more grade B embryos in the EmbryoScope^TM^ than in the standard embryology group (36.8 ± 8.5 vs. 7.4 ± 4.1 %, *P* = 0.01), while numbers of embryos with poor quality (grade C, not suitable for transfer or cryopreservation) were similar (7.3 ± 5.7 vs. 11.0 ± 4.7 %, *P* = 0.62).

**Table 5:** Embryo quality comparison in study Part B

Embryo grades	EmbryoScope^TM^ (*n* = 36)	Standard (*n* = 40)	*P* value
Grade A (%)	55.8 ± 6.4	81.2 ± 4.1	0.005
Grade B (%)	36.8 ± 8.5	7.7 ± 4.1	0.01
Grade C (%)	7.3 ± 5.7	11.0 ± 4.7	0.62

*N* number of oocyte donors, *n* number of embryos

These findings suggest that, though the total number of usable embryos (Grade A + Grade B) was not affected by the two culture systems, standard embryo culture generated significantly better culture results since approximately 25 % of all embryos in the EmbryoScope^TM^ system ended up one grade below those handeled in standard embryology.

Part B did not allow for evaluation of pregnancy rates because patients were transferred good quality embryos in combination from both culture systems.

#### Investigation id EmbryoSlide™

Suspecting the conical shape of the culture dishes (EmbryoSlide™) to be the reason for the inferior performance of the EmbryoScope^TM^, we then prospectively tested another 132 consecutively produced embryos from 10 egg donor cycles by alternating them in standard embryo culture for 3 days (up to cleavage stage) in an open incubator between EmbryoSlide™ (*n* = 68) and our center’s standard culture dish (*n* = 64).

Table 6 demonstrates no difference in embryo quality between both culture dishes. These findings suggest that differences in embryo quality between the EmbryoScope™ and standard embryology in Part B was not caused by variations in embryo culture dishes between the two systems but, likely, reflected the culture environment of the EmbryoScope™.

**Table 6:** Embryo quality after culture in EmbryoSlildes™ and standard culture dishes in standard open system incubators

Embryo grades	EmbryoSlide (*n* = 68)	Standard dishe (*n* = 64)	*P* value
Grade A (%)	79.7 ± 3.8	80.1 ± 2.4	0.83
Grade B (%)	9.8 ± 3.1	13.1 ± 3.8	0.44
Grade C (%)	10.3 ± 4.4	7.5 ± 3.0	0.61

*N* number of oocyte donors, *n* number of embryos

## Discussion

Universal efficacy and safety of embryo selection via use of TLI embryo culture systems in human IVF laboratories has so far not been established [11]. Moreover, maybe even more importantly, whether these closed culture systems are clinically equally effective in different patient populations has never before been investigated.

Embryos of good and poor prognosis or of younger and older women are known to behave differently during in vitro culture. The aim of this study was, therefore, twofold: (i) to assess whether a TLI system achieves similar IVF outcomes to standard manual embryology; and (ii) whether the efficacy of a TLI system is the same in better and poorer prognosis patients.

Our center does not change laboratory pratices without prior assessments of non-inferiority and, hopefully, determination of superiority. Before purchasing a closed incubation system, we, therefore, contacted different manufacturers in attempts to perform a prospectively randomized pilot study to assess how such a system would perform in our center’s highly adversely selected patient population. Only one, the manufacturer of the EmbryoScope™, graciously agreed to provide us with a loaner instrument for a 3-months study period. Intarmural research funds were used to pay for installation of the instrument, staff training and supply costs. The study only commenced, once the manufacturer was confident that our center’s embryology staff was competent in using the instrument.

Limited time availability of the system resticted the number of patients we were able to investigate. Here presented conclusions, therefore, should be considered as preliminary, even though they in important aspects are based on statistically significant results.

### Prospective randomization of poor prognosis patients (Part A)

Part A prospectively investigated randomized patients with relatively poor prognosis between tradional embryology and the EmbryoScope™. When our investigation was initiated, the instrument had been investigated in only one RCT, involving very favorably selected patients That study reported the instrument to marginally improve IVF outcomes (27). As effectiveness of some IVF interventions differs between good-, intermediate- and poor-prognosis patients [31], we felt the need to assess its efficacy in our patient population before committing to a purchase. Ages, FSH and AMH levels of here investigated patients (Table 1) reflect the poor outcome prognosis of our patients in comparison to the Spanish study by Rubio et al. [27].

That in contrast to their study no outcome benefits from the EmbryScope™ were seen in our investigation is, therefore, noteworthy. Our findings may suggest that closed incubation systems in different patient populations may demonstrate different degrees of efficacy. The small size and, therefore, inadequate power of our study, however, does not preclude a Type 2 error in failing to demonstrate an outcome difference in Part A of this study. Significant outcome differences may only become apparent with larger patient numbers. Since observed implantation and pregnancy rates actually trended toward traditional embryology (Table 3), a potential outcome benefit from the instrument over standard embryology, however, appears unlikely.

No differences (ie, no improvements) in embryo development with TLI systems have been reported before [19–21, 28–32]. One study, indeed, suggested increased miscarriage rates with use of a TLI system [26]. As also suggested by Racowsky et al. [11], whether TLI systems really offer outcome benefits over standard embryology, therefore, remains questionable,

### Embryology staff time

In promotional efforts, manufactueres of TLI systems also have claimed that these systems save embryology staff time. In this first investigation of such a claim claim, as Table 4 demonstrates, the EmbryoScope™ almost doubled embryo observation times in comparison to standard embryology. Trying to determine the cause(s), we discovered that the instrument required repeated adjustment of focus because initial monitor images were not as clear as with manual inverted microscopy. Embryo scoring was also more challenging since embryos tended to migrate toward the sides of wells, as also reported by Park et al. [26]. The EmbryoScope™, thus, does not appear to save embryology staffing time, and, indeed, may increase staffing needs.

### Randomization of embryos in best prognosis patients (Part B)

Part B of this study was intended to assess the instrument in “best” prognosis patients. In using young oocyte donors, large oocyte yields allowed open randomization of embryos between EmbryoScope™ and standard embryology in place of patient randomization. In the previously noted Spanish RCT, oocyte donors represented almost half of the patient population [27]. The intent, therefore, was to investigate performance of the TLI system in best-prognosis patients, even exceeding the favorable patient selection of the Spanish study.

Since based on the Spanish study we expected improvements in IVF outcomes, we were surprised to observe the opposite. We were further surprised by the statistical power of observed outcome differences, even though small numbers call for caution in interpretation: Despite the relative small study size (Table 5), the EmbryoScope™ produced significantly fewer Grade A and, therefore, significantly more Grade B embryos than standard embryology, suggesting a potentially negative impact of the TLI system on embryo quality.

Observing these rather surprising results raised the question whether staff members operating the EmbryoScope™ had been sufficiently trained. We, therefore, reviewed embryology staffing records, and found that the embryologists handling the instrument during Parts A and B of here reported study were exactly the same. Insufficient staff training, therefore, only unlikey explains the findings of this study.

Our suspicion then fell on the culture dish of the EmbryoScope™, called the EmbryoSlide™. It is a single-use, sterile culture dish, especially designed for the EmbryoScope^TM^ incubator. Each EmbryoSlide™ holds up to 12 embryos, each cultured individually in droplets of 25 μl media. They, therefore, are cultured in relative low density and not grouped together. In contrast, conventional dishes, as utilized at our center, culture up to 5 embryos in 50 μl droplets.

Higher embryo density group culture has been reported to benefit embryo development [16, 33], possibly the consequence of one or more factors produced by embryos, which can stimulate embryo development [34]. We, therefore, parallel cultured alternating donor embryos in EmbryoSlides™ and standard embryo culture dishes in the same incubation environmentone (Table 6). As the table demonstrates, this additional prospective evaluation by day-3 of culture demonstrated no difference in embryo quality between both embryo culture dishes. The EmbryoSlide™, therefore, apparently was not the cause of our observation.

This left us with no established cause for our observation, and the conclusion that the incubation envrironment in the EmbryoScope™, likely, was inferior to our center’s standard embryo culture environment. Such an explanation is not farfetched: Several physical factors can impact final embryo development during in vitro culture, including incubation volume/embryo density, temperature/pH and light as well as shear stress from mechanical motion [34]. Even minimal changes in temperature during culture (away from 37 °C) for short time periods can result in unrecoverable damage by hurting the stability of the oocye/embryo spindle [35, 36]. The pH of medium also plays a crucial role in embryo culture [34], while regulation of pH mostly relies on the carbon dioxide (CO_2_) concentration.

The user manual of the EmbryoScope™, notes that the instrument is equipped with unique temperature controls, characterized by direct heat transfer to individual media-filled wells. Temperature is alleged to be virtually unchanged by opening the chamber (<0.2 °C) when adding or removing embryos. Recovery of CO_2_ concentrations is alleged to occur in less than 5 min and of O_2_ in less than 15 min after closing of chamber. During this study, we. indeed, based on the built-in monitoring software, did not note temperature and/or CO_2_ concentration changes of any significance.

It has been suggested that prolonged light exposure required for time-lapse photography may negatively affect embryos [14, 20, 37]. The EmbryoScope^TM^, however, uses long wave length light of lower intensities (red light, 635 nm) than the light used in our embryology laboratory during standard embryo assessments under a microscope (15 % <550 nm). There also are currently no experimental data in the literature to support any negative effects of greater light exposure on embryos with use of the EmbryoScope^TM^ [20, 26].

All of this leaves only one likely explanation for our findings: The EmbryoScope^TM^ contains a microscope, built into a compact incubator. During image capture, the microscope is fixed, while the tray and culture dish move slightly to focus each single well. Since in our study, images were taken every 10 min, embryos during three days of culture had to move at least 380 times. Though this embryo motion is very gentle and mild, the impact of possible sheer stress cannot be ruled out as a potential cause for observed declines in embryo quality [34].

The effect may be statistically more apparent in best prognosis patients because even a relative small percentage loss in pregnancy in such a population may be statistically more apparent. This also would explain why the reported improvement in outcomes in the Spanish study [27], which included only a little less than half of best prognosis patients (ie, egg donors) was only marginal. If our here laid out assumptions are correct, had the Spanish study included even more oocyte donors (ie, good prognosis patients), it too, may no longer have demonstrated marginally better IVF cycle outcomes with the TLI system or might even have drifted into the negative.

Mouse models that investigated the effects of mechanical vibration induced shear stress on embryo development, demonstrating decreased morula and blastocyst formation [38], caused by phosphorylating mitogen-activated protein kinase (MAPK) 8/9 [39]. Similar phosphorylation of MAPK 8/9 was never observed in control embryo or in vivo cultured embryos.

## Conclusions

All of above noted considerations suggest that the most likely explanation for the inferior performance of the EmbryoScope™ in good prognosis donors is increased exposure of embryos to shear stress. This is, however, as of this point still a hypothesis, which requires experimental confirmation. If confirmed, this negative finding may not be applicable to other TLI systems with different camera systems.

Because of its limited size, as already noted, here presented data have to be interpreted with caution. We also have to acknowledge that we followed embryo outcomes in this study only to cleavage-stage (day-3), while TLI systems are primarily meant to be used to culture embryos to blastocyst stage (days 5/6). Though unlikely, it is possible that TLI systems affect prolonged embryo culture more favorably. Since blastocyst stage culture really only benefits good prognosis patients [31] this, at best represents only a double-edged-sword since the opposite may actually also be true, and benefits of a TLI system by day-3 may be overestimated.

This study, however, clearly demonstrates the need for larger, well designed prospectively randomized studies before TLI systems are placed into human embryology laboratories for routine IVF care. It is also important to point out that here reported study results cannot be generalized since they reflect the quality of our center’s manual embryology. Outcome comparisons with manual embryology may obviously differ at different embryology laboratories.

If properly maintained, likely the quintessential advantage of a TLI system is stability of its performance. Less competent or less stable manual embryology may, therefore, indeed benefit from systems like the EmbryoScope™, while superior manual embryology can, at least in good prognosis patients, likely outperform TLI systems. A TLI like the EmbryoScope™ may, thus, actually improve IVF outcomes in programs with unstable embryology by stabilizing performance; yet, in a program with excellent embryology, effects may be the opposite.

In full disclosure, based on here reported study results, our center for the time being is foregoing the purchase of TLI systems.

## Abbreviations

AMH, anti-Müllerian hormone; COCs, oocyte-cumulus complexes; FDA, Food and Drug Administration; FSH, follicle-stimulating hormone; GnRHa, gonadotropin releasing hormone agonist; hCG, human chorionic gonadotopin; hMG, human menopausal gonadotropin; HSA, human serum albumin; ICSI, intracytoplasmic sperm injection; IRB, institutional review board; IVF, in vitro fertilization; RCT, randomized controlled trial; SEM, standard error of the mean; TLI, time-lapse imaging
